# Embryonic Origin and Remodeling of the Urinary and Digestive Outlets

**DOI:** 10.1371/journal.pone.0055587

**Published:** 2013-02-04

**Authors:** Chen Wang, JingYing Wang, Joseph G. Borer, Xue Li

**Affiliations:** 1 Department of Urology, Boston Children's Hospital, Boston, Massachusetts, United States of America; 2 Department of Surgery and Pathology, Harvard Medical School, Boston, Massachusetts, United States of America; 3 Harvard Stem Cell Institute, Cambridge, Massachusetts, United States of America; Pennington Biomedical Research Center/LSU, United States of America

## Abstract

Separating digestive and urinary outlets is a critical step during mammalian embryogenesis. However, the natural history of these structures is poorly studied, and little is known about their embryonic origin. Here, we show that peri-cloacal mesenchymal (PCM) progenitors are the major source of these structures. Surprisingly, PCM progenitors also contribute to perineum, a structural barrier separating the urinary and digestive tracts, suggesting a potential role of PCM progenitors in establishing independent urinary and digestive outlets. We demonstrate that *Six1* and *Six2* are complementarily but asymmetrically expressed in the PCM progenitors. Deletion of these genes results in decreased cell survival and proliferation, and consequently in agenesis of the perineum and severe hypoplasia of the genital tubercle. Together, these findings suggest that PCM progenitors are the unexpected source of perineum and genital tubercle, and establish a basic framework for investigating normal and abnormal development of anorectal and genitourinary structures.

## Introduction

Partitioning of a hollow structure is one of the most fundamental remodeling processes during embryogenesis. For example, a single tube of cardiac outflow tract is divided into pulmonary and aortic trunks - a vital step that ensures separation of oxygen-rich and oxygen-depleted blood circulations. Cloaca, the most caudal end of the hindgut, is a common primordial structure of both digestive and urinary outlets. Developmental anomalies involving cloaca remodeling are among the most common forms of human birth defects. However, cloaca morphogenesis and remodeling of digestive and urinary outlets have received little attention and are poorly understood.

A prevailing textbook model indicates that a putative urorectal septum divides the cloaca along the dorsoventral axis. The dorsal compartment forms the digestive outlet including rectum and anus, while the ventral urogenital sinus undergoes complex transformation to form bladder, urethra as well as related reproductive organs. More than a century ago, Rathke suggested that fusion of the bilateral longitudinal folds (Rathke's fold) led to formation of the urorectal septum [1]. In this model, two bilateral ridges fuse like a zipper moving caudally to divide the cloaca into two compartments. This concept is supported by Retterer in the 1890s [2] and recently by investigators including Hynes and Fraher [3]. However, lack of essential evidence to support tissue fusion, including localized apoptosis and/or epithelial-to-mesenchymal transition, casts serious doubt on the model [4]–[8]. Indeed, Tourneaux proposed an alternative interpretation, and suggested that the urorectal septum is a coronally-oriented wedge of mesenchyme, known as the Tourneux's fold [9], which divides cloaca like a theater curtain dropping in a rostral to caudal direction. In contrast to these two urorectal septum-based models, van der Putte liked the cloaca to a “tubular structure” that is “increasingly more bent toward the surface” [5], [6]. Based on this interpretation, an entirely different ventral displacement model was put forward, which suggested that a disproportionate growth of ventral relative to dorsal cloacal mesenchyme transforms instead of divides the cloaca into the urogenital and digestive compartments. It is unclear, however, how such transformation leads to the separation of the urinary and digestive tracts.

Despite the differences among these interpretations, all models suggest that a discrete population of mesenchymal progenitors is critical for dividing the cloaca. However, a paucity of molecular and cell biological studies of cloacal mesenchymal progenitors hinders our ability to reconcile the controversies of the aforementioned models. The perineum is the diamond-shape area superficial to the pelvic diaphragm and bordered by the pubic arch, ischial tuberosities and coccyx [6]. The term “perineum” is also used for the restricted area between the anus and the urethral orifice, we refer this region as the “midline epithelium of the perineum” to avoid confusion. Since the perineum is the physical barrier that separates urinary and digestive outlets, a better understanding of its embryonic origin would have an important implication in cloacal morphogenesis. According to the classic Rathke's fold and the Tourneux's fold models, the putative urorectal septum consisting of intra-cloacal mesenchyme (ICM) gives rise to the perineum [1], [2]. The ventral displacement model, on the other hand, does not explicitly address the nature of progenitors that contribute to the perineum [5]. Genetic lineage tracing studies of cloacal epithelial cells demonstrate that midline surface epithelium of the perineum has an endodermal origin [10]. This observation implies that the ICM progenitors are the source of perineum, and indirectly supports the cloacal septum-based models. However, a direct genetic fate mapping analysis of the peri-cloacal mesenchyme (PCM) progenitors instead suggests that PCM are the major source of the perineum [11]. Therefore, the central issue of embryonic origin of the perineum remains to be elucidated.

In this study, we use an inducible genetic fate-mapping approach to interrogate PCM lineages; and demonstrate that the PCM progenitors contribute directly to the perineal stromal tissue. We show for the first time the complementary and asymmetrical expression patterns, as well as their lineage distribution patterns, of *Six1* and *Six2* in PCM progenitors. Deletion of these two genes results in a decreased PCM progenitor cell survival and proliferation, and consequently severe genital tubercle hypoplasia and perineum agenesis. Thus, PCM is an unexpected source of perineum, which is essential for formation and remodeling of cloaca and urogenital structures. Taken together, these findings suggest that a process reminiscent to vascular occlusion results in a partitioning of cloaca, and provide a basic framework for investigating cellular and molecular mechanisms of urinary and digestive outlet development.

## Results

### Asymmetric and complementary expression patterns of *Six1* and *Six2* in PCM progenitors

**Figure 1:**
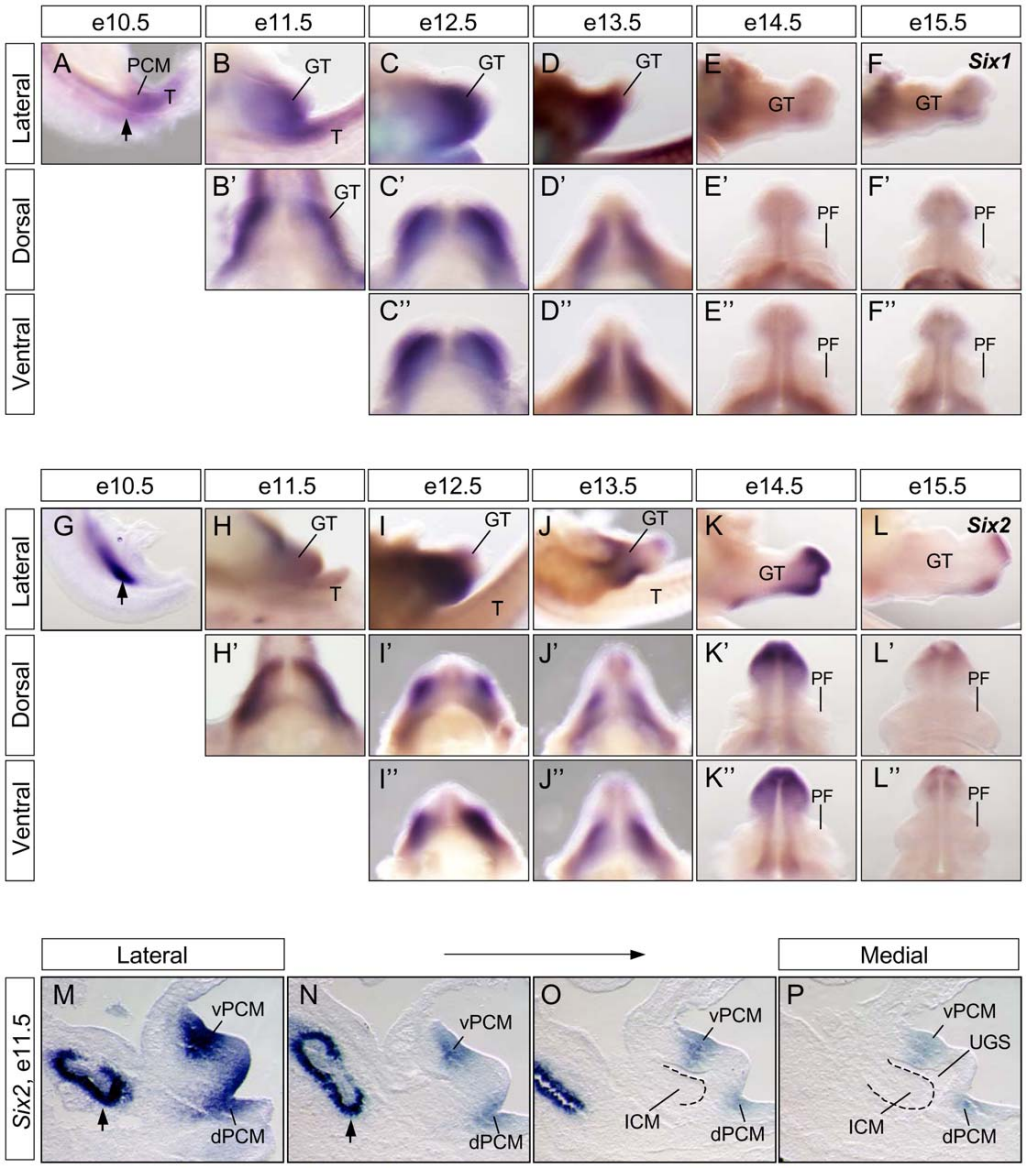
Dynamic expression patterns of *Six1* and *Six2* during urogenital development. Whole-mount *in situ* hybridization of staged embryos, using *Six1-* (**A–F**) and *Six2-* (**G–L**) specific probes, were visualized laterally (**A–L**), dorsally (**B′–L′**) and ventrally (**C″–L″**). (**M–P**). *Six2 in situ* hybridization was performed on a series of e11.5 sagittal sections. C, cloaca; GT, genital tubercle; ICM, intra-cloacal mesenchyme; PCM, peri-cloacal mesenchyme; dPCM, dorsal PCM; vPCM, ventral PCM; PF, preputial fold; T, tail; arrow, metanephric mesenchyme; UGS, urogenital sinus.

Among six different members of *Six1-*family transcription factors, the high degree of similarity between *Six1* and *Six2* suggests that they may share similar function *in vivo* [12], [13]. We have shown that *Six1* is highly expressed in the PCM progenitors with a dorsal-to-ventral gradient, and that Six1 is required for normal urinary tract development [11]. To begin to characterize the potential function of *Six2*, we first compared its dynamic expression pattern with *Six1* (Fig. 1). *Six1* transcripts were detected in PCM cells as early as e10.5 (Fig. 1A). Its expression was maintained in genital mesenchyme between e11.5–e13.5 (Figs. 1B–D). At later stages (e14.5 and e15.5), *Six1* expression was significantly reduced and restricted to mesenchyme adjacent to the urethral plate and became undetectable in the preputial fold at e14.5 (Figs. 1E and F).

*Six1* was weakly expressed in metanephric mesenchyme (MM) but highly expressed in PCM at e10.5. On the other hand, *Six2* was enriched in MM but was hardly detectable in PCM at this stage (Fig. 1G, arrow). A day later, at e11.5, both genes were highly expressed in the genital swellings (Figs. 1B and H). At later stages, *Six2* was strongly expressed in mesenchymal cells surrounding the urethral plate at e13.5 but was significantly down regulated at e15.5 (Figs. 1J–L). Similar to *Six1*, *Six2* expression was diminished in the preputial fold (Figs. 1K and L). To highlight a spatial distribution pattern of *Six2* at the critical period of cloacal morphogenesis at e11.5, we performed RNA *in situ* hybridization experiments on serial adjacent sagittal sections. *Six2* appeared to be expressed in all PCM progenitors (Fig. 1M). However, its transcripts were enriched in the ventral PCM (vPCM) and reduced in the dorsal PCM (dPCM) (Fig. 1M–P). This asymmetric expression pattern is in contrast to *Six1*, *which* is highly expressed in the dPCM [11]. In addition, *Six2* was absent from the urorectal septum, which consists primarily of the ICM progenitors (Figs. 1O and P). Thus *Six1* and *Six2* have asymmetric, yet complementary, expression patterns in PCM progenitors, with *Six1* enriched dorsally and *Six2* ventrally. Both genes are absent from ICM cells.

### *Six2*-expressing PCM progenitors contribute to urogenital tissues including the perineum

**Figure 2:**
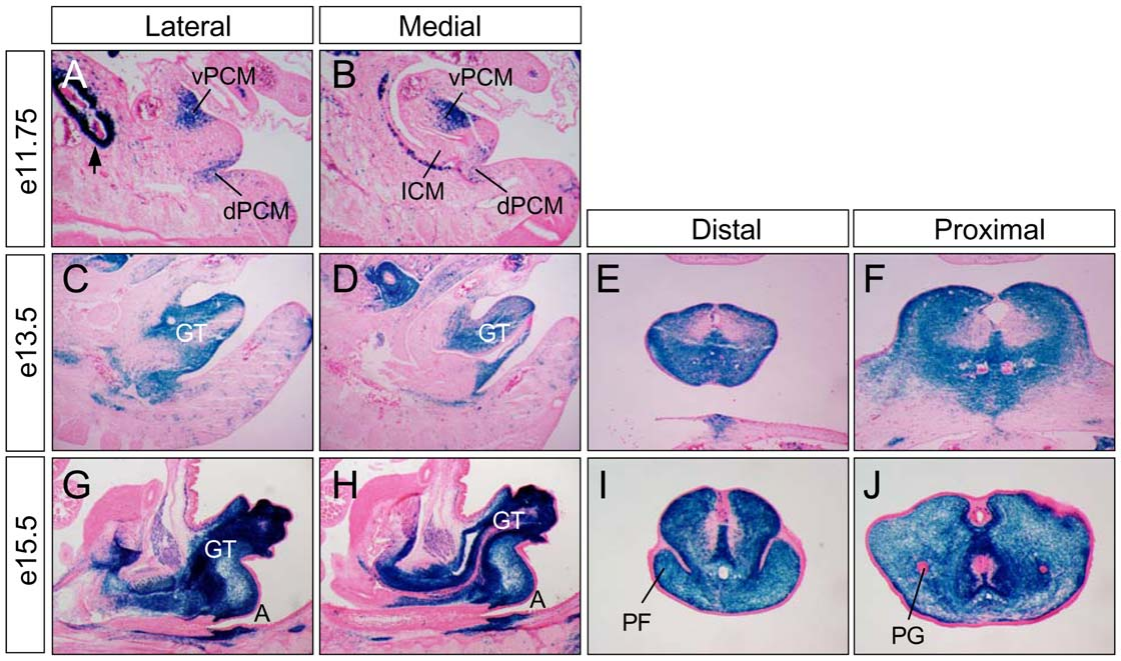
A genetic fate map of *Six2-*expressing PCM progenitors. X-gal staining (blue) of sagittal (**A–D**, **G** and **H**) and cross (**E**, **F**, **I**, **J**) sections from e11.75, e13.5 and e15.5 *Six2^GC/+^;R26R^LacZ^* double heterozygous embryos. All sections were counterstained with eosin (pink). A, anus; PG, preputial gland; see Figure 1 for more abbreviations.

The restricted *Six2* expression pattern in PCM cells provided a unique opportunity to interrogate lineage distribution patterns of PCM progenitors during development, as well as remodeling of urinary and digestive outlets. We first performed a genetic fate mapping analysis using a *Six2^GC^* mouse line (Fig. 2). The eGFP and Cre fusion gene (*GC*) replaces and fully recapitulates the endogenous *Six2* gene expression pattern since the same targeting strategy were used to generate other *Six2* mutant alleles, including *Six2^GCE^* allele [14]. The GC fusion protein has a constitutively-active, site-specific Cre recombinase activity that is able to turn on expression of a LacZ reporter, *R26R-lacZ* (*R26R^lacZ^*) [15]. Consequently, *Six2-*expressing progenitors and their progenies are selectively and permanently labeled by *lacZ* in *Six2^GC/+^;R26R^lacZ/+^* double heterozygous mice. We analyzed these embryos at three developmental stages before (e11.75) and after (e13.5) cloacal septation, and during perineum formation (e15.5) (Fig. 2). Sagittal and cross sections of genital tubercles were assayed for *lacZ* gene activity, a surrogate of *Six2* lineages. At e11.75, *lacZ^+^* cells were detected in the metanephric mesenchyme, vPCM, dPCM, and to a much less extent, the urethral plate and anorectal epithelial cells. No *lacZ^+^* cells were observed in the genital tubercle ectodermal epithelial cell layer (Figs. 2A and B). At e13.5 and e15.5, the majority, if not all, urogenital mesenchyme including the perineal stromal and preputial fold tissues were *lacZ^+^* cells (Figs. 2C–J). Few *lacZ^+^* cells at the urethral plate and anorectal epithelium were observed at e13.5 and e15.5 (Figs. 2C–J). In addition, mesenchymal cells surrounding the anal canal were all *lacZ*-positive (Fig. 2G and H). Thus, *Six2^+^* PCM progenitor cell lineages contribute to most, if not all, anogenital mesenchymal tissues.

**Figure 3:**
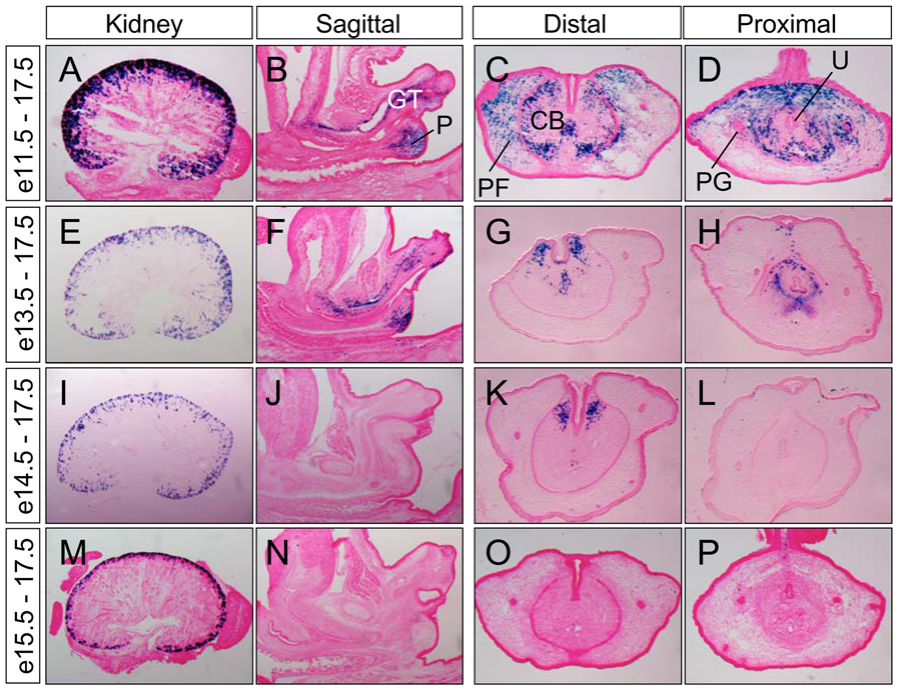
An inducible genetic fate map of *Six2-*expressing PCM progenitors. Double *Six2^GCE/+^*;*R26R^LacZ^* pregnant females were treated with a single dose of tamoxifen at e11.5, e13.5, e14.5 and e15.5, and all embryos were collected and analyzed at e17.5 with X-gal staining (blue). (**A**, **E**, **I** and **M**) kidney sections; (**B–D**, **F–H**, **J–L** and **N–P**) urogenital sections. CB, prospective corporal body; GT, genital tubercle; P, perineum; PF, preputial fold; PG, preputial gland; U, urethra.

We next sought to determine when PCM progenitors are committed to these distinct tissues. Toward this end, we used another *Six2^GCE^* mouse line, which expresses a tamoxifen-inducible eGFP and CreER (GCE) fusion protein, to map the fate of *Six2-*expressing PCM progenitors [14]. A single dose of tamoxifen was used to treat females pregnant with *Six2^GCE/+^;R26R^lacZ/+^* double heterozygous embryos at e11.5, e13.5, e14.5 and e15.5, and these embryos were analyzed at e17.5 for *lacZ* reporter gene activity. Since *Six2* is strongly expressed in renal progenitors (Fig. 1), we used the kidney as an indicator of efficient tamoxifen-induced Cre recombination (Figs. 3A, E, I and M). Tamoxifen treatment at e11.5 resulted in extensive *lacZ^+^* cells in the kidney; as expected, progressively fewer *lacZ^+^* cells were detected in kidneys that were treated with tamoxifen at later stages (Fig. 3A, E, I and M). We next analyzed the spatiotemporal distribution patterns of *lacZ^+^* cells in urogenital tissues from these same embryos. Tamoxifen treatment at e11.5, a stage in which *Six2* was strongly expressed in PCM but absent from ICM cells (Figs. 1M–P), resulted in abundant *lacZ^+^* cells that were broadly distributed in the perineum, preputial fold and the prospective corporal body (Figs. 3B–D). Though fewer in number, a similar distribution pattern of *lacZ^+^* cells was observed when tamoxifen was administrated at e13.5 (Figs. 3F–H). In contrast, tamoxifen injections at later stages (e14.5 and e15.5) resulted in *lacZ^+^* cells only at the distal genital tubercle region, near the urethral plate (Figs. 3J–L, 3N–P and data not shown). No *lacZ^+^* cell was detected in the perineum in these embryos. Together, results from these constitutive and inducible genetic fate-mapping analyses demonstrate that the PCM progenitors are the major source of the perineum, and that these progenitors are committed to the fate of the perineum as early as e11.5 prior to separation of the urinary and digestive outflow tracts.

### *Six1* and *Six2* have redundant functions in PCM progenitors

**Figure 4:**
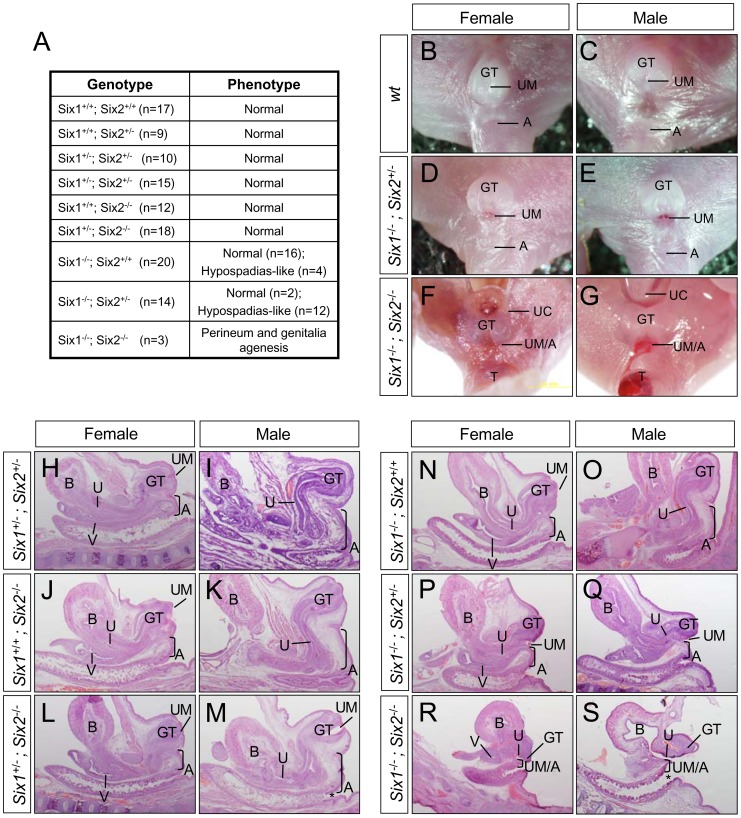
Genital urinary and anorectal defects of *Six1;Six2* compound mutants. (**A**) A table of urogenital phenotypes of *Six1;Six2* compound mutants. (**B**–**G**) Gross ventral views of external urogenital structures. (**H**–**S**) Hematoxylin and eosin (H&E) staining of midline sagittal sections of urogenital structures from newborn pups. A, anus; B, bladder; GT, genital tubercle; T, tail; UM, urethral meatus; UC, umbilical cord; U, urethra; V, vagina.

Mouse mutants lacking either *Six1* or *Six2* die at birth due to renal agenesis defects [12], [14], [16]–[18]. All of *Six2^−/−^* (n = 12) and *Six1^+/−^;Six2^−/−^* (n = 18) mutants had grossly normal genital tubercle and anal structures [11] (Fig. 4A and data not shown). Only a small percentage (20%, n = 20) of *Six1^−/−^* embryos had a displacement of the urethral meatus at a ventral and proximal region of the external genitalia, resembling a hypospadias-like phenotype (Fig. 4A and data not shown) [19]–[21]. Loss of one allele in the *Six2* in *Six1^−/−^* mutant background (*Six1^−/−^;Six2^+/−^*) (n = 14) increased penetrance of the hypospadias-like phenotype to 85.7% (Figs. 4A, D and E). Mutant genital tubercles were overall smaller than wild type littermate controls. In addition, the urethral meatus were displaced at the base of external genitalia (Figs. 4D and E). Loss of both genes (*Six1^−/−^;Six2^−/−^*, n = 3) resulted in agenesis of the perineum and severe hypoplastic external genitalia (Figs. 4A, F and G). Thus, *Six1* and *Six2* have redundant and essential functions in PCM progenitors during perineum and genital tubercle formation.

To better understand urogenital and anorectal defects of *Six1;Six2* compound mutants, we performed histological analysis of serial sagittal sections from newborn male and female pups (Figs. 4H–S). Perineal stromal tissue, which separates urinary and digestive tracts, was apparent and indicated by the anogenital distance in both wild type male and female pups (Figs. 4H–S, bracket). The same tissue in the *Six1^−/−^;Six2^+/−^* mutant was hypoplastic, and the anogenital distance was significantly reduced (Fig. 4P and Q). Consistent with these gross defects, the mutant genital tubercles were hypoplastic. The *Six1* and *Six2* double null mutants exhibited a severe agenesis defect since the genital tubercle and the perineum were nearly absent (Fig. 4R and S). In addition, the anal canal of the double null mutants was absent, resulting in a direct exposure of rectum epithelium (Fig 4, compare asterisk in M and S). Together, these findings suggest that *Six1* and *Six2* are required for the development of both digestive and urinary outlets.

### Survival and proliferation of PCM progenitors depend on *Six1* and *Six2*

**Figure 5:**
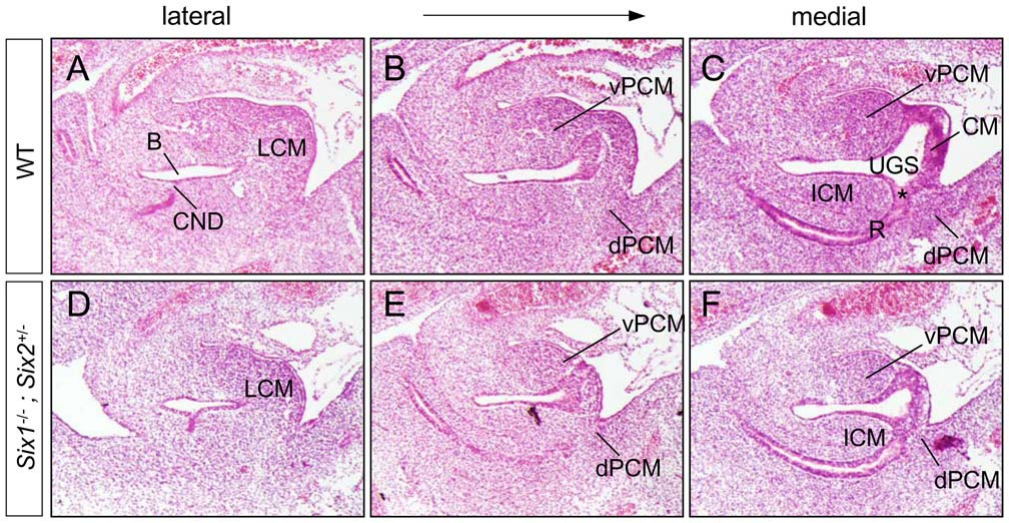
Hypoplastic genital tubercles of *Six1;Six2* compound mutants at e11.5. Hematoxylin and eosin (H&E) staining of the serial sagittal sections of e11.5 urogenital structure. Asterisk, juxtaposition of ICM, dPCM and the cloacal membrane (CM); B, bladder; CND, common nephric duct; lPCM, lateral PCM; R, rectum; see Figure 1 for more abbreviations.

Because of the rarity of obtaining double null mutants, we used *Six1^−/−^;Six2^+/−^* compound mutants to further characterize primary defects of digestive and urinary outlets during early embryogenesis. In wild type embryos, three populations of mesenchymal cells were apparent at e11.5 along midline sagittal sections, the ventral vPCM, the dorsal dPCM and the internal ICM (Fig. 5). The caudal side of the cloaca was covered by the cloacal membrane, which was a composite of endoderm and ectoderm epithelia but devoid of any mesenchyme. At this stage, the distal end of ICM was juxtapositioned but not fused with dPCM and the cloacal membrane (Fig. 5C, asterisk), the likely site of the future anal canal. This unique juxtaposition separated the urogenital sinus and rectum, thereby serving as the first sign of separation between the urinary and the digestive tract (Fig. 5C). Asymmetric growth of these mesenchymal cells was likely involved in remodeling of the urogenital sinus to form the genital tubercle and the anal canal. In *Six1^−/−^;Six2^+/−^* mutants, the relative position of the cloacal mesenchyme, the cloacal membrane, and the unique juxtaposition were maintained (Fig. 5F). However, it was apparent that both the dPCM and the vPCM were hypoplastic, and that the size of the mutant genital tubercle was significantly smaller (Fig. 5D–F, and data not shown). These observations suggest that *Six1* and *Six2* may control the growth and/or expansion of these tissues.

**Figure 6:**
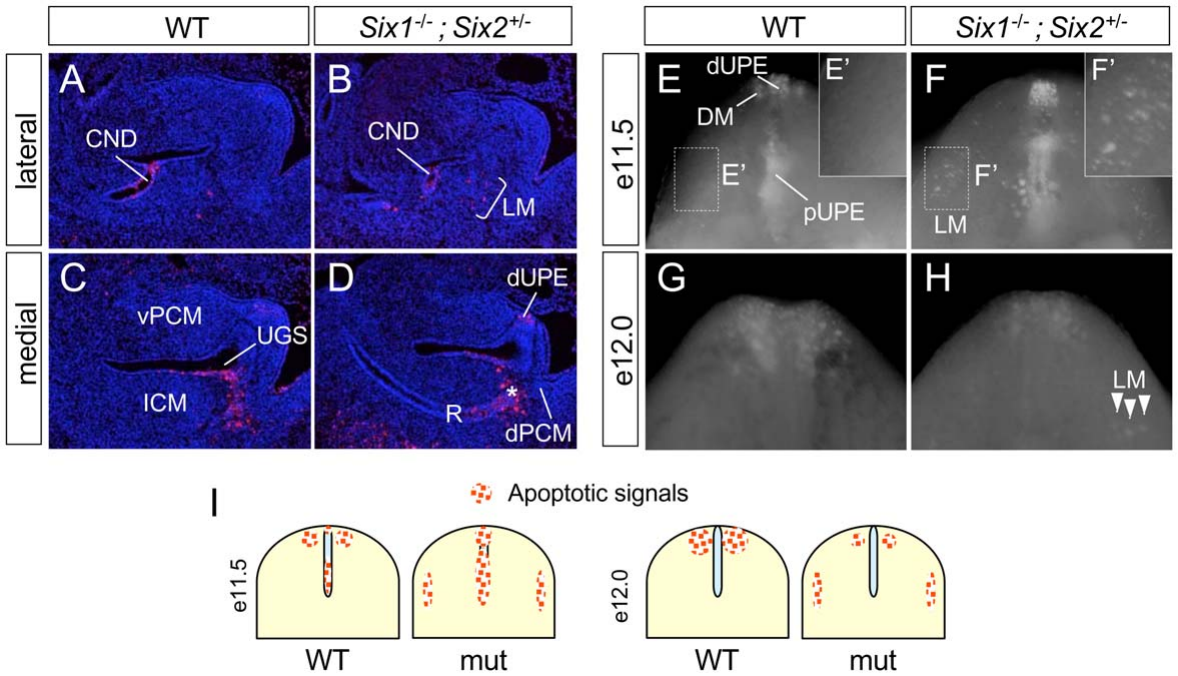
*Six1;Six2* compound mutant genital tubercles have aberrant apoptosis patterns. (**A–D**) Comparable levels of serial of sagittal sections were stained with TUNEL for apoptotic cells (red). All sections were counter stained with DAPI (blue). (**E–H**) Ventral views of genital tubercles (GTs) stained with Lysotracker® for apoptotic cells (white dots). Apoptotic cells were observed in e11.5 control GTs within distal urethral plate epithelia (dUPE), proximal urethral plate epithelia (pUPE), distal mesenchymal (DM) (E). In *Six1^−/−^;Six2^+/−^* mutant, Lysotracker® signals were enhanced within dUPE and pUPE but not detectable within DM (F). Ectopic apoptotic cells in the lateral mesenchyme region (LM) was observed (E′, insert). At later stages, apoptotic cells were reduced in *Six1^−/−^;Six2^+/−^* mutants at e12.0 within DM (G and H). Ectopic cell death in LM persisted at e12.0 (arrowheads, H). (**I**) Schematic representations of dynamic changes of apoptosis patterns in control and mutants. CND, common nephric duct; distal mesenchymal (DM); LM, lateral mesenchyme; dUPE, distal urethral plate epithelium; pUPE; proximal urethral plate epithelium; R, rectum; see Fig. 1 for more abbreviations.

Since *Six1* is required for the survival of renal and cardiac progenitors [12], [16], [22], we first used TUNEL assays to determine if survival of the PCM progenitors depended on *Six1* and *Six2* (Fig. 6). Similar levels of apoptotic cells were detected in the common nephric duct in littermate controls and *Six1^−/−^;Six2^+/−^* mutants (Fig. 6A and B), which therefore functioned as an internal control [23]. Apoptotic endoderm and mesenchyme were detected at the juxtaposition at e11.5, as expected [24], [25], however, more apoptotic cells were present in *Six1^−/−^;Six2^+/−^* mutants (Fig. 6D, asterisk). Enhanced apoptosis was also observed in the distal urethral plate epithelium in the mutants (Fig. 6D). In addition, an ectopic TUNEL signal was detected in genital mesenchyme at lateral sections (Fig. 6B).

To investigate the spatial distribution pattern of the apoptotic cells, we next used a vital dye LysoTracker Red to examine apoptosis pattern of whole mount embryos at e11.5 (Fig. 6E–H). Three distinct apoptotic domains - distal urethral plate, distal mesenchymal region and proximal urethral plate - were apparent with the LysoTracker Red signal (Fig. 6E). Deletions of *Six1* and *Six2* caused an increase of apoptosis in the urethral plate (Fig. 6F). Consistent with the TUNEL staining findings, ectopic cell death was detected in the lateral mesenchymal region in mutants (compare Fig. 6E′ to F′). This ectopic increase of cell death persisted at e12.0 (arrowhead in Fig. 6H). Surprisingly, the mutant genital tubercle underwent less cell death in the distal mesenchymal region at e11.5 and e12.0 (Figs. 6E, F and I). As illustrated in Fig. 6I, deletion of *Six1* and *Six2* alters both the apoptosis level and pattern, changes which may contribute to the hypoplastic defect of the genital tubercle.

**Figure 7:**
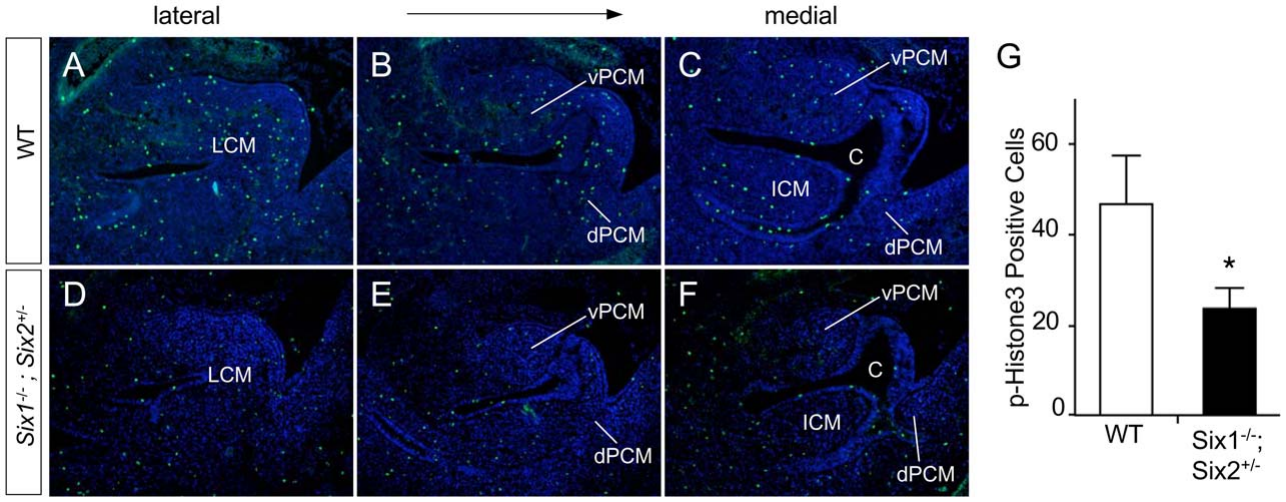
*Six1* and *Six2* are required for proliferation of PCM progenitors. (**A–F**) Phospho-histone H3 staining (p-HH3, green) of proliferating cells using a series of sagittal sections at e11.5. (**G**) Quantification of p-HH3 staining results.

We also examined the cell proliferation status of the *Six1^−/−^;Six2^+/−^* mutants by staining serial embryonic sections with a p-HH3 antibody [11], a mitosis marker of cell cycle. Sections with similar overall anatomic features were selected for this study (Fig. 7). A total of 8 cryostat sections that encompassed genital tubercle were stained with the p-HH3 antibody. Using the shape of cloaca endoderm lining as a guide, proliferating cells from dPCM, vPCM and ICM were counted and compared. The total number of p-HH3 positive cells was significantly lower in *Six1^−/−^;Six2^+/−^* mutants than in wild type littermate controls (Fig. 7G). Thus, the normal proliferation of PCM progenitors depends on *Six1* and *Six2*. Collectively, these data indicate that *Six1* and *Six2* are required for proliferation and survival of the PCM progenitors, which may contribute directly to the observed severe hypoplastic phenotype (Fig. 4R and S).

### *Six1* and *Six2* coordinate expression of critical signal molecules

**Figure 8:**
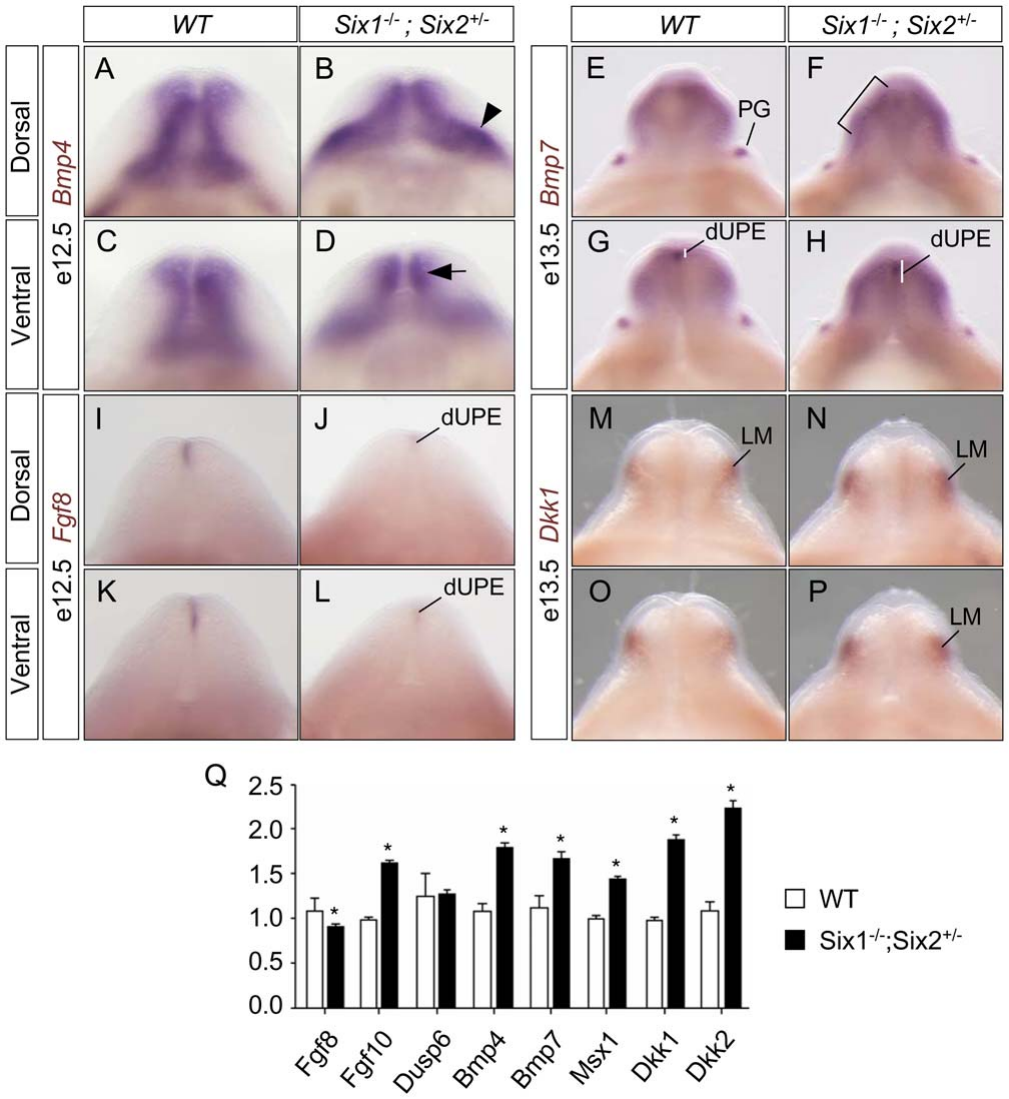
Aberrant expression of signal molecules during urogenital development in *Six1;Six2* compound mutants. (**A–P**) Whole mount *in situ* hybridization of genital tubercles using gene-specific probes of *Bmp4*, *Bmp7*, *Fgf8*, and *Dkk1*. (**Q**) Real time quantitative PCR analysis of gene expression levels of micro-dissected genital tubercle tissue at e11.5. Arrow, ventral distal mesenchyme; arrowhead, dorsal lateral mesenchyme; bracket, enhanced expression of *Bmp7*; dUPE, distal urethral plate epithelium; LM, lateral mesenchyme; PG, preputial gland.

Activation of the Bone morphogenetic protein *(Bmp)* signaling pathway inhibits genital outgrowth and induces apoptosis [26], [27]. Mouse mutants without *Noggin* (an antagonist of *Bmp* signaling pathway) display hypoplastic genitalia, while *Bmp* receptor Ia (*BmprIa*) mutants have overgrowth and reduced cell death, resulting in hyperplastic genitalia [27]. *Bmp7* mutants have imperforate anus and genital tubercle defects [28]. Deletion of *Eya1*, a transcription coactivator of *Six1* and *Six2*, results in increased *Bmp* signaling [11]. To examine whether *Six1* and *Six2* were required for expression of candidate genes that are important for genital tubercle growth and patterning, we therefore first examined the expression pattern of *Bmp4* and *Bmp7* (Figs. 8A–D, 8E–H). We found that *Bmp4* expression was enhanced in dorsal lateral mesenchyme (arrowhead in Fig. 8B) and ventral distal mesenchyme (arrow in Fig. 8D) of *Six1^−/−^;Six2^+/−^* mutants. *Bmp7* expression in the distal urethral plate epithelium (dUPE) was expanded and extended proximally toward the base of the tubercle in mutants, and its expression in genital tubercle mesenchyme was increased (Figs. 8F and H). Upregulation of *Bmp4* and *Bmp7* was further confirmed by real time quantitative PCR (qPCR) using microdissected genital tubercle at e11.5 (Fig. 8Q). Furthermore, the expression of Bmp4 downstream target genes *Msx1* [11], [27], were significantly upregulated in *Six1^−/−^;Six2^+/−^* mutants (Fig. 8Q), suggesting that *Bmp* signaling was enhanced.

The canonical *Wnt/ß-catenin* signal pathway is critical for the normal development of urogenital structures [29], [30]. Interaction between androgen and Wnt/ß-catenin signal pathway promotes formation of male external genitalia [31]. *Dkk1* and *Dkk2* are potent inhibitors of the *Wnt/ß-catenin* signal pathway [32]–[35]. In addition, *Dkk1* is a downstream effector of *Bmp* signaling, and together they promote apoptosis [36]. Because *Six1^−/−^;Six2^+/−^* mutants displayed increased *Bmp* signaling (Fig. 8A–H, Q) and apoptosis (Fig. 6), we therefore examined the expression level of *Dkk1* and *Dkk2*. At e13.5, *Dkk1* transcripts were detected in mesenchymal cells lateral to the urethral plate, and expression of Dkk1 was slightly upregulated in the *Six1^−/−^;Six2^+/−^* mutants (Figs 8M–P). Consistently, both *Dkk1* and *Dkk2* genes were significantly upregulated in the mutant genital tubercle at e11.5, based on a quantitative PCR analysis of micro-dissected tissues (Fig 8Q).

*Six1* is required for Fibroblast growth factor *(Fgf8)* expression during cardiac and craniofacial development [22]. Exogenous *Fgf8* promotes genital tubercle outgrowth in organ cultures [37], and its expression in the distal urethral plate depends on both *Shh* and *Wnt/ß-catenin* signaling pathways [29], [30], [38], [39]. However, conditional deletion of *Fgf8* has no obvious genital tubercle defect [40]. On the other hand, a mutation in murine *Fgf10* results in a hypospadias-like phenotype [41]. We detected reduced expression of *Fgf8* in *Six1^−/−^;Six2^+/−^* mutants at e12.5 (Figs. 8I–L), but increased expression of *Fgf10* (Fig. 8Q), suggesting that downregulation of *Fgf8* might be compensated by upregulation of *Fgf10*. Indeed, expression of dual specificity protein phosphatase 6 (*Dusp6*), which is downstream of the *Fgf* signaling pathway [11], [40], was not affected (Fig. 8Q).

Taken together, these candidate gene expression analyses suggest that deletions of both *Six1* and *Six2* disrupt dynamic expression patterns of several critical signal molecules required for normal development of urogenital structures.

## Discussion

Our findings uncover that PCM progenitors are the unexpected source of perineum and urogenital organs. We show for the first time that *Six1* and *Six2* are asymmetric and complementarily expressed in the PCM progenitors, where they are required for proliferation and survival of these progenitors. These observations are suggestive that a process reminiscent to vascular occlusion underlies the partitioning of cloaca and remodeling of urogenital structures.

**Figure 9:**
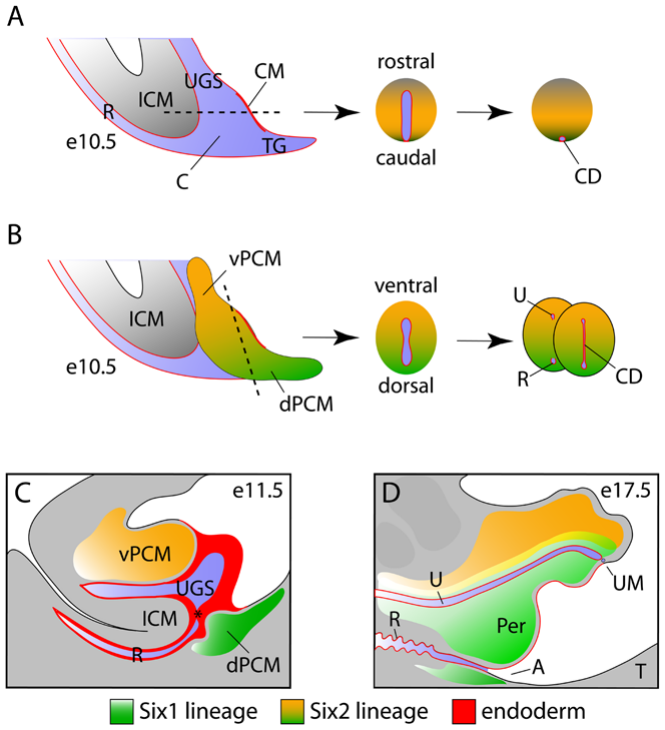
A working model: patterning of cloacal mesoderm leads to occlusion of the cloaca and outgrowth of the genital tubercle. (**A** and **B**) Asymmetric growth and patterning along the rostrocaudal axis (A) and dorsoventral axis (B) causes occlusion and division of cloaca into urinary and digestive tracts. The process also displaces the cloacal duct (CD), remnant of the cloacal epithelium, to the surface of perineum as a thin epithelial lining. (**C** and **D**) Midline sagittal diagrams of genital tubercle at e11.5 (C) and e17.5 (D). Continuous growth of peri-cloacal mesenchyme leads to remodeling and opening of the anal canal and urethra, and of the digestive and urinary outlets, respectively. Peri-cloaca mesenchymal progenitors contribute to most, if not all, stromal tissues of genital tubercle and perineum. Asterisk, juxtaposition of ICM, dPCM and the cloacal membrane; A, anus; C, cloaca; CD, cloacal duct; CM, cloacal membrane; ICM, intro-cloacal mesenchyme; PCM; peri-cloacal mesenchyme; dPCM, dorsal PCM; vPCM, ventral PCM; Per, perineum; R, rectum; T, tail; TG, tail gut; U, urethra; UGS, urogenital sinus; UM, urethral meatus.

Asymmetric growth of mesenchyme is the major driving force that transforms cloaca into urinary and digestive tracts (Fig. 9). Therefore, patterning of the cloacal mesoderm is a central issue of cloaca morphogenesis. Along the rostrocaudal axis, cloaca is surrounded by mesenchyme at the rostral ICM cells and lateral PCM cells but not the caudal cloacal membrane, which is devoid of mesenchyme (Fig. 9A). Thus, an intrinsic asymmetry is established because of the absence of mesenchyme in the cloacal membrane. A rapid increase in both PCM and ICM cells occludes the cloacal cavity and separates the hindgut (rectum and anal canal) and urogenital sinus (bladder and urethra). The process also pushes the cloacal duct, the remnant of cloaca, caudally towards the surface of the perineum. Consequently, independent digestive and urinary tracts are established, and the cloaca duct persists at the midline surface of perineum epithelium.

Unlike the intrinsic asymmetry of rostrocaudal axis, cloaca is surrounded at all sides by the PCM progenitors along the dorsoventral axis (Fig. 9B and C). It is not immediately clear how asymmetric gene expression and growth along dorsoventral axes are established. An intriguing observation is the high levels of apoptosis at the dPCM and tail gut region (Fig. 6) [24], [25]. This localized cell death likely retards growth of the dPCM, thereby causing asymmetric growth along the dorsoventral axis and a ventral shift of the cloacal membrane, as proposed by van der Putte [6]. Asymmetric expression patterns of *Six1* and *Six2* suggest that PCM is indeed patterned along the dorsoventral axis, as *Six1* is highly enriched in the dPCM [11] while *Six2* is enriched in vPCM (Fig. 1M–P). Consistently, Six1-positive lineages are predominantly localized at the ventral side of the genital tubercle (Fig. 9) [11]. We have also shown that *Six1* and *Six2* coordinately control proliferation and survival of PCM progenitors, potentially through candidate signal molecules (Fig. 8), and that genetic deletion of *Six1* and *Six2* results in agenesis of the perineum and severe hypoplastic genitalia. These data suggest that patterning along the dorsoventral axis is required for completion of cloacal division, as well as outgrowth and patterning of the genital tubercle.

*Shh* is expressed in the cloacal endoderm and is required for all stages of genitourinary tract development [30], [38], [39]. *Shh* signaling controls cell cycle kinetics of mesenchyme [42]. It is worth noting that *Six6*, a homology of *Six1*, is directly involved in modulating cell cycle of retinal progenitor [43]. *Shh* is maintained in *Six1* and *Six2* compound mutants (data not shown) and *Eya1* mutant [11], raising a possibility that *Shh* maybe an upstream regulator. A key future question would be to understand intrinsic and extrinsic mechanism underlying the asymmetric growth and patterning of the cloacal mesenchyme.

The proposed cloacal occlusion model is supported by the unexpected origin of the perineum discovered here and previously [10], [11]. Seifert et al., reported previously that the midline epithelium of the perineum has an endodermal origin [10]. Of the various models put forth, the cloaca occlusion model best accounts for the observations of the shape (a narrow line) and asymmetric positioning (midline caudal surface) of the endoderm remnant (Fig. 9A and B). As illustrated in Figure 9A, occlusion of the cloaca results in displacement of the cloaca duct and formation of the perineum. On the other hand, the Rathke's fold model predict that any surviving endodermal cells would be randomly distributed and embedded in the perineum stromal layer [1], [2]. The Tourneux's fold model [9], as well as the transformation model [5], would have predicted that the cloacal remnant is distribution broadly at the surface of the perineum. Both *Six1* and *Six2* are expressed in the PCM, but not ICM progenitors. Interestingly, *Six1-* and *Six2-* positive cell lineages contribute directly to perineum tissue. Furthermore, inducible genetic fate mapping demonstrates, for the first time, that *Six2* expression in PCM progenitor contributes to the perineal stromal tissue, as early as e11.5. While the occlusion model does not exclude the possibility that ICM progenitors might also contribute to the perineum, these observations suggest that PCM progenitors are involved in perineum formation. Indeed, *Six1* and *Six2* double null mutants exhibit perineum agenesis defects.

Taking together, we conclude that cloacal mesoderm progenitors are central to the separation of the urinary and digestive tracts, as well as to the outgrowth and patterning of genital tubercle. We postulate that asymmetric growth causes narrowing of cloaca at the location marked by the cloacal membrane. The endodermal remnant is pushed to the midline epithelial surface of perineum between the anus and urethral meatus. We now refer to the morphogenesis event of the cloaca as “cloacal occlusion”. Since the term septation has between used in many developmental contexts, and implies explicitly the existence of a septum, which is still a mater of speculation during cloacal morphogenesis, we therefore prefer the use of the cloacal occlusion to describe the mechanism of cloacal division. This model provides a basic framework, i.e. patterning along dorsoventral and rostrocaudal axes, with which to investigate normal and abnormal development of the anorectal and genitourinary structures.

## Materials and Methods

### Mice

All animal studies were performed according to protocols reviewed and approved by the institutional animal care and use committee at the Children's Hospital Boston. *Six1* [12], *Six2^GCE/+^*, *Six2^GC/+^* [14], *R26R^lacZ^* [15] mice have previously been reported. Genotyping of the mice was performed as described. For temporal induction of Cre recombination, tamoxifen (Sigma, T5648) was dissolved in sesame oil (Sigma, S3547) and administrated by intraperitioneal (IP) injection (50 g/kg body weight).

### Histology, Immunohistochemistry and in situ hybridization

Embryos were fixed in 4% paraformaldehyde (PFA), embedded in OCT, and frozen sections prepared at a thickness of 12 um. H&E staining were performed by standard procedures. β-galactosidase activity was detected with previously described methods and counter stained with eosin [11], [12], [22], [43]. Whole-mount and section *in situ* hybridization were performed as described previously [11], [22]. To label proliferating cells, e11.5-staged embryos were dissected, fixed in 4% paraformaldehyde for 2 hours at 4°C, and then sectioned at 14 um. Embryo sections were incubated with anti-pH3 (Upstate) at a 1∶200 dilution, as previously described [11], [22]. The number of p-HH3+ cells was averaged from 6 sections per embryo.

### TUNEL and Lysotracker Red® Staining

Both terminal deoxynucleotidyl transferase nick end labeling (TUNEL) (Roche) and Lysotracker® (Invitrogen) staining were performed according to the manufacturer's protocol and described previously [11]. Briefly, embryos were dissected and stained with 5 uM lysotracker® in PBS at 37°C for 30 min. Embryos were then washed in PBS several times prior to PFA fixation. Microdissected genital tubercles were imaged using an Olympus SZX16 fluorescence dissection microscope equipped with a DP71 digital camera. Cryostat sections were used for TUNEL assays.

### Quantitative real-time PCR analysis

Genital tubercle tissue of e11.5 embryos was micro-dissected and snap-frozen on a dry ice/ethanol bath. RNA was purified based on manufacturer's protocols (Qiagen RNAeasy mini). cDNA was synthesized using the Stratagene Accuscript™ High Fidelity 1st strand cDNA synthesis (Agilent Technologies) using 200 ng of total RNA. Relative gene expression levels were normalized to an α-actin internal control, and analyzed using SYBR Green Master Mix (Affymetrix) on an ABI-7500 detector (Applied BioSystems). The following oligos were used: α-actin F: TCG TCG ACA ACG GCT CCG GCA TGT; α-actin R: CCA GCC AGG TCC AGA CGC AGG AT; Bmp4 F: GCC GAG CCA ACA CTG TGA GGA; Bmp4 R: GAT GCT GCT GAG GTT GAA GAG G; Bmp7 F: GGA GCG ATT TGA CAA CGA GAC C; Bmp7 R: AGT GGT TGC TGG TGG CTG TGA T; Msx1 F: AGG ACT CCT CAA GCT GCC AGA A; Msx1 R: CGG TTG GTC TTG TGC TTG CGT A; Fgf8 F: GGG AAG CTA ATT GCC AAG AG; Fgf8 R: TGT ACC AGC CCT CGT ACT TG; Fgf10 F: GCC ACC AAC TGC TCTT CTT C; Fgf10 R: CTG ACC TTG CCG TTC TTC TC; Dkk1 F: ATA TCC CAG AAG AAC CAC ACT G; Dkk1 R: CTT TCC GTT TGT GCT TGG TG; Dkk2 F: GCA TCC TCA CCC CAC ATA TC; Dkk2 R: CGA GCA CAA CAA AAC CCA TC; Dusp6 F: CTC GGA TCA CTG GAG CCA AAA C; Dusp6 R: TCT GCA TGA GGT ACG CCA CTG T.
